# Supplementary material for: Neutralising SARS-CoV-2 RBD-specific antibodies persist for at least six months independently of symptoms in adults
Source: Commun Med (Lond). 2021 Jul 14;1:13. doi: 10.1038/s43856-021-00012-4 (PMC9037317; doi:10.1038/s43856-021-00012-4)
Supplement: Supplementary file 1 — Supplementary Materials [file 43856_2021_12_MOESM1_ESM.pdf]

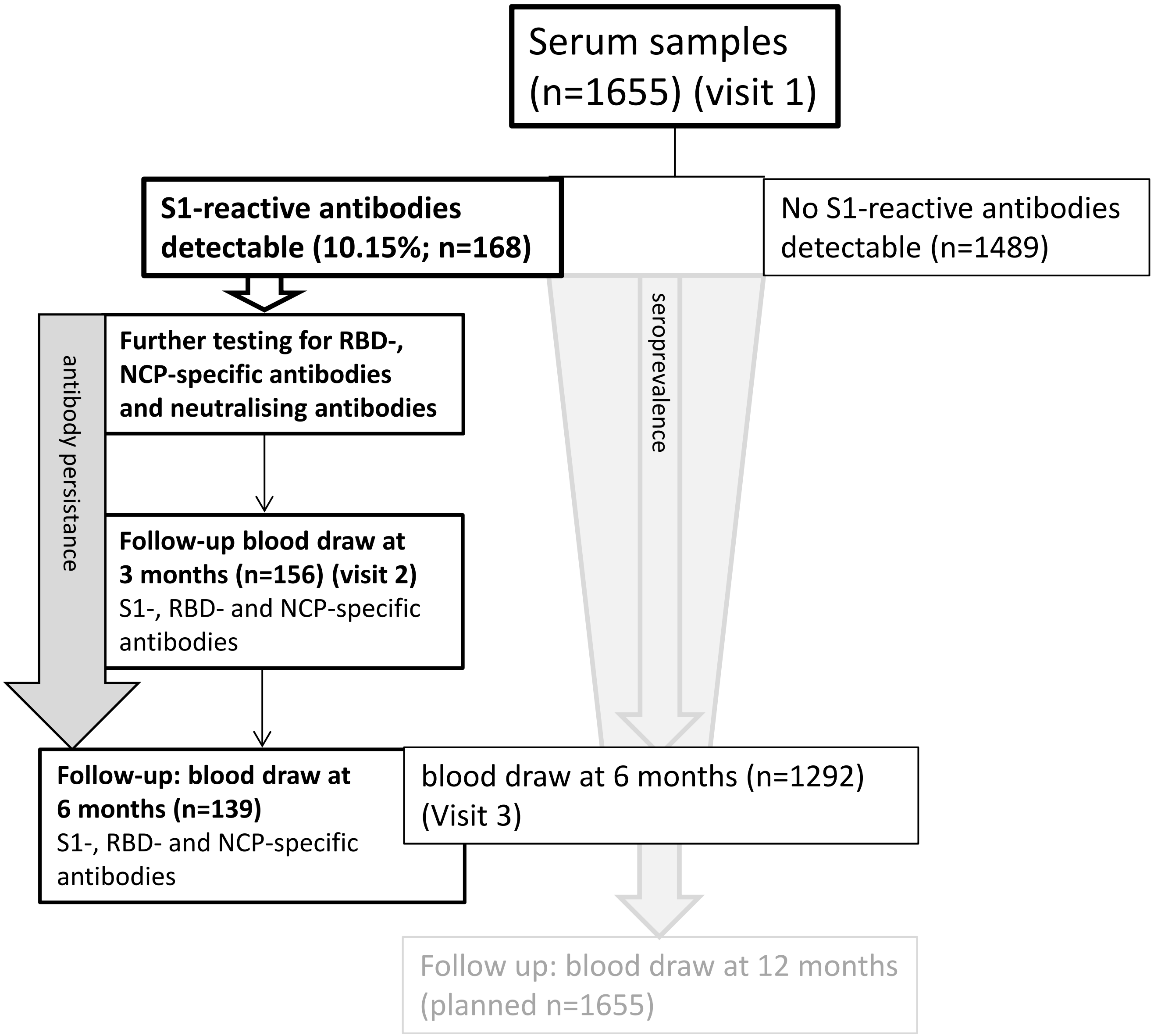

Suppl. Figure 1: Flow chart of participants throughout six months. Serum samples with S1-reactive antibodies at the initial blood draw and at six months were further analysed for RBD- and NCP-specific antibodies. S1 subunit of spike protein (S1), receptor binding domain (RBD), nucleocapsid (NCP).

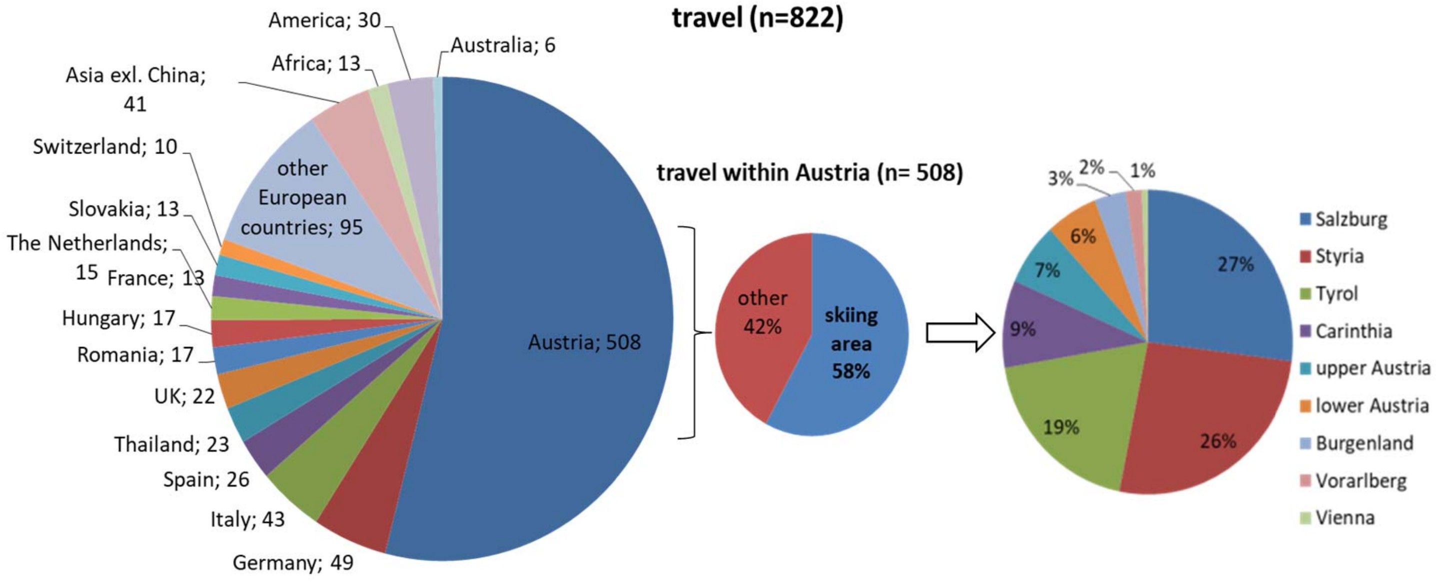

Suppl Figure 2: Travel history within three months before the first blood draw as reported from all participants at the day of the initial blood draw (day 0). Multiple answers were possible.

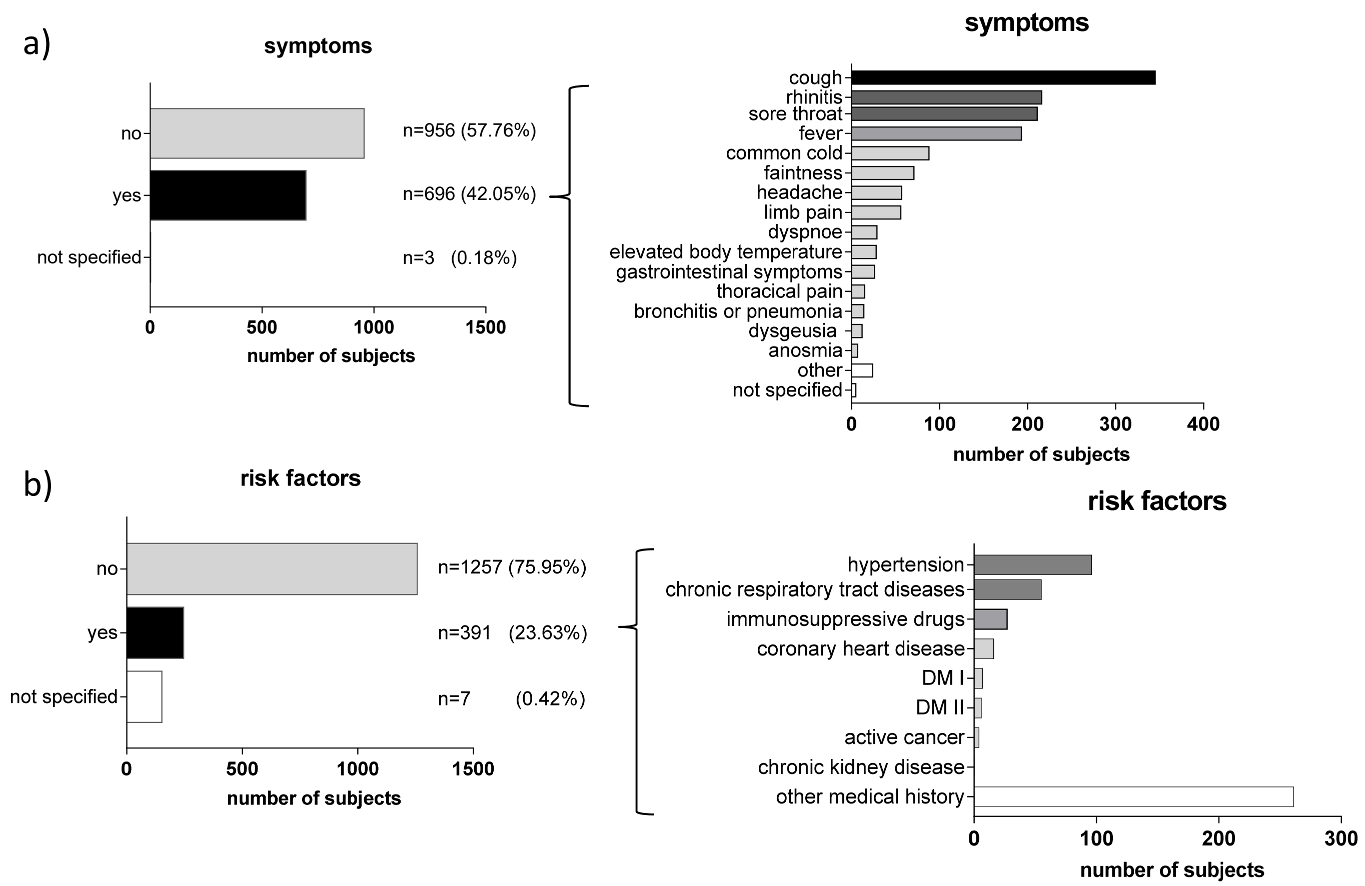

Suppl Figure 3: Overall occurence of symptoms (a) within three months to the initial blood draw as well as presence of risk factors (b) the total study population that were reported at the day of the initial blood draw (day 0). Multiple answers were possible

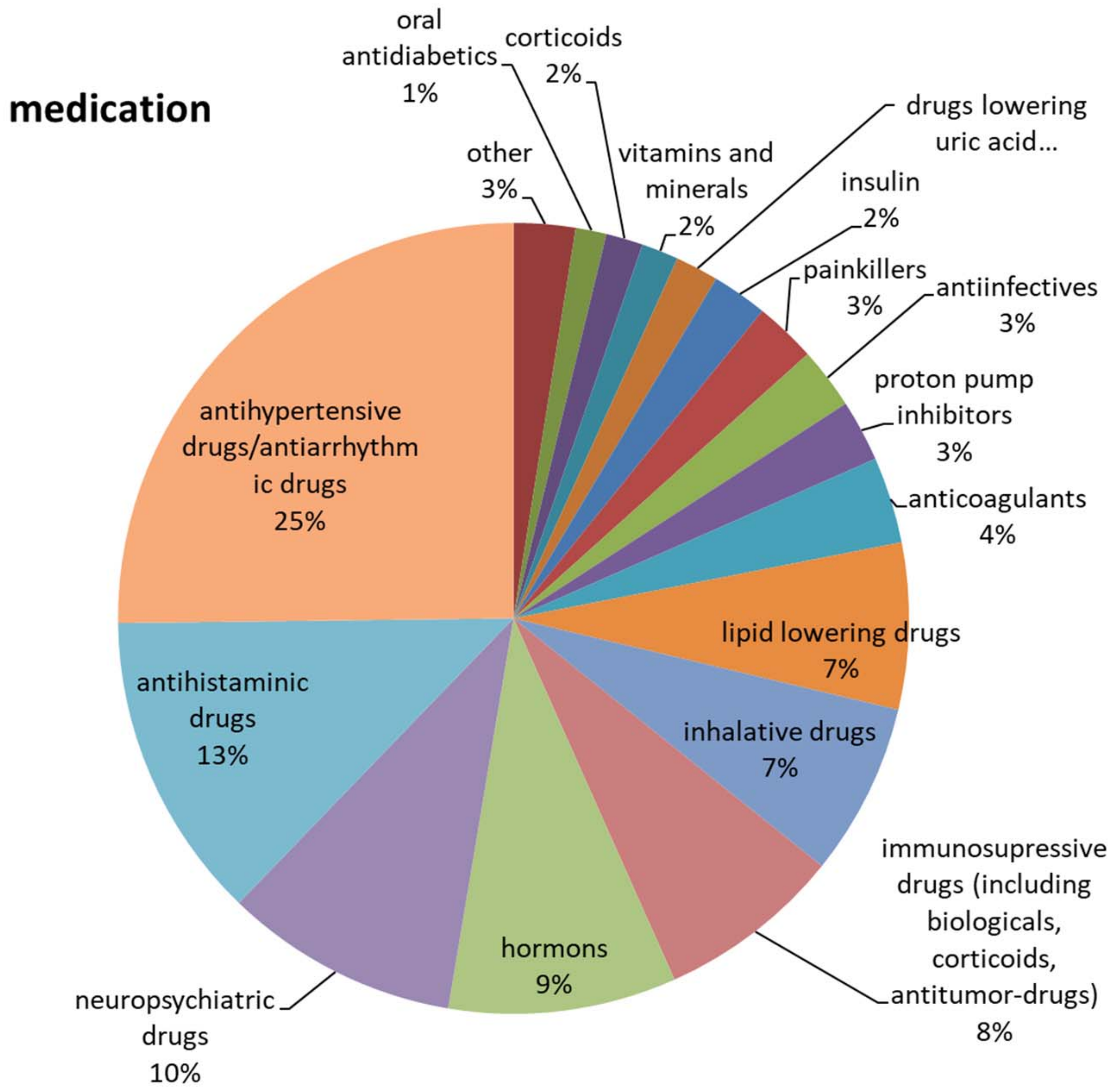

Suppl Figure 4: Regular intake of medication as reported at the initial blood draw (day 0) from all participants. Multiple answers were possible.

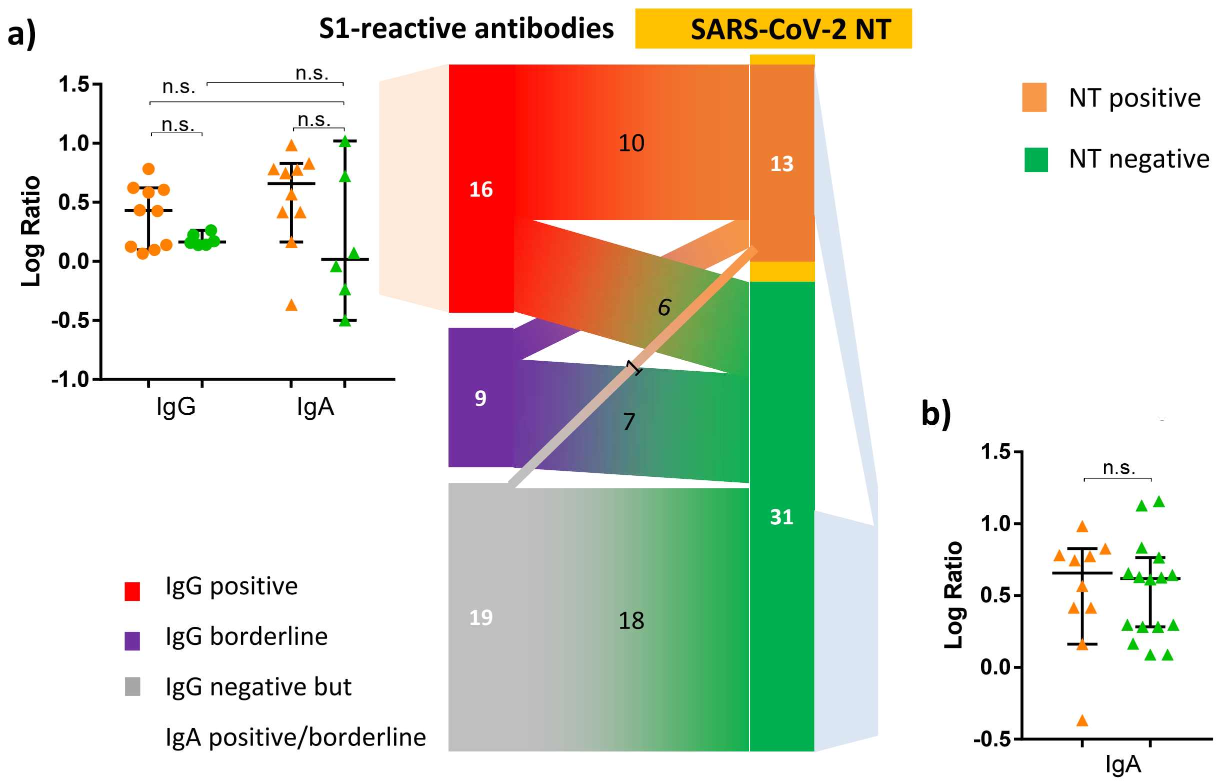

Suppl Figure 5: Comparison of S1-reactive IgG and IgA antibody levels in participants according to the neutralisation test (NT) result (positive = orange (n=10); negative = green (n=6)) in participants with a positive (red) S1-specific IgG result (n=16) (a) and the comparison of the S1-reactive IgA ratio levels between those with a positive (n=10) and a negative (n=16) neutralisation test result at the first blood draw (day 0) (b). S1-reactive antibody ratios presented after logarithmic transformation. Violet: borderline values for S1-reactive antibodies, grey: negative S1-reactive IgG but IgA positive/borderline. S1 subunit of spike protein (S1), neutralisation test (NT). Line and bars represent median +/- 95%CI.
