# Supplementary material for: Neutralising SARS-CoV-2 RBD-specific antibodies persist for at least six months independently of symptoms in adults
Source: Commun Med (Lond). 2021 Jul 14;1:13. doi: 10.1038/s43856-021-00012-4 (PMC9037317; doi:10.1038/s43856-021-00012-4)
Supplement: Supplementary file 3 — Description of Additional Supplementary Files [file 43856_2021_12_MOESM3_ESM.pdf]

Description of Additional Supplementary Files

File Name: Supplementary Data 1

Description: Data used to generate the charts and graphs in the main figures of the manuscript
