# Supplementary material for: Neutralising SARS-CoV-2 RBD-specific antibodies persist for at least six months independently of symptoms in adults
Source: Commun Med (Lond). 2021 Jul 14;1:13. doi: 10.1038/s43856-021-00012-4 (PMC9037317; doi:10.1038/s43856-021-00012-4)
Supplement: Supplementary file 5 — Reporting Summary [file 43856_2021_12_MOESM5_ESM.pdf]

nature research

Corresponding author(s): Prof. Ursula Wiedermann-Schmidt

Last updated by author(s): 16.06.2021

Reporting Summary

Nature Research wishes to improve the reproducibility of the work that we publish. This form provides structure for consistency and transparency in reporting. For further information on Nature Research policies, see our Editorial Policies and the Editorial Policy Checklist.

Statistics

For all statistical analyses, confirm that the following items are present in the figure legend, table legend, main text, or Methods section.

n/a	Confirmed	
☐	☒	The exact sample size (n) for each experimental group/condition, given as a discrete number and unit of measurement
☐	☒	A statement on whether measurements were taken from distinct samples or whether the same sample was measured repeatedly
☐	☒	The statistical test(s) used AND whether they are one- or two-sided<br>*Only common tests should be described solely by name; describe more complex techniques in the Methods section.*
☐	☒	A description of all covariates tested
☐	☒	A description of any assumptions or corrections, such as tests of normality and adjustment for multiple comparisons
☐	☒	A full description of the statistical parameters including central tendency (e.g. means) or other basic estimates (e.g. regression coefficient) AND variation (e.g. standard deviation) or associated estimates of uncertainty (e.g. confidence intervals)
☐	☒	For null hypothesis testing, the test statistic (e.g. F, t, r) with confidence intervals, effect sizes, degrees of freedom and P value noted<br>*Give P values as exact values whenever suitable.*
☒	☐	For Bayesian analysis, information on the choice of priors and Markov chain Monte Carlo settings
☒	☐	For hierarchical and complex designs, identification of the appropriate level for tests and full reporting of outcomes
☒	☐	Estimates of effect sizes (e.g. Cohen's d, Pearson's r), indicating how they were calculated

Our web collection on statistics for biologists contains articles on many of the points above.

Software and code

Policy information about availability of computer code

Data collection	data were collected in excel (Version 2016)
Data analysis	standard methods of data analysis were applied

For manuscripts utilizing custom algorithms or software that are central to the research but not yet described in published literature, software must be made available to editors and reviewers. We strongly encourage code deposition in a community repository (e.g. GitHub). See the Nature Research guidelines for submitting code & software for further information.

Data

Policy information about availability of data

All manuscripts must include a data availability statement. This statement should provide the following information, where applicable:

- Accession codes, unique identifiers, or web links for publicly available datasets
- A list of figures that have associated raw data
- A description of any restrictions on data availability

The datasets generated during and/or analysed during the current study are available from the corresponding author (UW also principal investigator) on reasonable request and after confirmation by the ethics committee. Data used to generate the charts and graphs in the main figures of the manuscript are provided in a supplementary data file.

Field-specific reporting

Please select the one below that is the best fit for your research. If you are not sure, read the appropriate sections before making your selection.

☒ Life sciences ☐ Behavioural & social sciences ☐ Ecological, evolutionary & environmental sciences

For a reference copy of the document with all sections, see nature.com/documents/nr-reporting-summary-flat.pdf

Life sciences study design

All studies must disclose on these points even when the disclosure is negative.

Sample size	Sample size was predetermined by the number of available blood samples. It was expected to compile data sets from approx. 800 employees continuing to work on-site with client contacts and some 700 home office workers. Based on the numbers of notified SARS-CoV-2 PCR-positive individuals in Vienna and an estimated number of 10 times the number of unreported cases, it was assumed that approx. 1% of the population had already had contact with the virus prior to the blood draw. Presuming that these figures would also apply to the employees included in the study, the effect size expressed as an odds ratio of 3 at a two-sided level of significance of 5% resulted in a statistical power of 77% to compare employees on-site and in home office (Fisher´s exact probability test).
Data exclusions	No data were excluded.
Replication	We used validated commercial assays for antibody measurement. Samples within the borderline range and with ratios close to the cut-off of 0.8 or 1.1 (value from 0.7 to 1.2) were repeated for confirmation in two independent tests and the geometric mean was used for the final result.
Randomization	Randomization was not relevant since it was an observational study
Blinding	Blinding was not relevant in this study as it was observational. However, observer blinding of lab personnel was applied.

Reporting for specific materials, systems and methods

We require information from authors about some types of materials, experimental systems and methods used in many studies. Here, indicate whether each material, system or method listed is relevant to your study. If you are not sure if a list item applies to your research, read the appropriate section before selecting a response.

Materials & experimental systems

n/a	Involved in the study	
☐	☒	Antibodies
☐	☒	Eukaryotic cell lines
☒	☐	Palaeontology and archaeology
☒	☐	Animals and other organisms
☐	☒	Human research participants
☐	☒	Clinical data
☒	☐	Dual use research of concern

Methods

n/a	Involved in the study	
☒	☐	ChIP-seq
☒	☐	Flow cytometry
☒	☐	MRI-based neuroimaging

Antibodies

Antibodies used	Anti-SARS CoV2 S, N and RBD antibody tests were applied using commercial kits.
Validation	Validation information is available in the information leaflet of the respective manufacturers. Positive and borderline S results were confirmed by testing for RBD and N antibodies in our study.

Eukaryotic cell lines

Policy information about cell lines

Cell line source(s)	Vero cells (ATCC CCL-81) sourced from European Collection of Authenticated Cell Cultures (84113001)
Authentication	Authenticated Cell Cultures (84113001)
Mycoplasma contamination	Verocells were tested for mycoplasma contamination and only negative cultures were used for the neutralisation test.
Commonly misidentified lines (See ICLAC register)	n.a.

Human research participants

Policy information about studies involving human research participants

Population characteristics	Participants were empoyees of a large company. They were either in home office or at their usual workplace.
Recruitment	Employees were informed about the study and the oportunity to get a test result from antibody testing. At precpecified dates employees that wanted to take part got additional verbal information and gave written informed consent.
Ethics oversight	Ethics Committee of the Medical University of Vienna

Note that full information on the approval of the study protocol must also be provided in the manuscript.

Clinical data

Policy information about clinical studies

All manuscripts should comply with the ICMJE guidelines for publication of clinical research and a completed CONSORT checklist must be included with all submissions.

Clinical trial registration	The study is not an interventional clinical trial and no registration was necessary.
Study protocol	A study protocol was submitted to the ethics committee
Data collection	Data were collected from April 2020 to November 2020.
Outcomes	Primary endpoint was the development of the seroprevalence over a 6 months observation period. Secondary endpoint was seroconversions of previously seronegative individuals at six months. Both parameters werde determined by SARS-CoV-2 specific antibody measurements.
